# Identification of *MiR-93-5p* Targeted Pathogenic Markers in Acute Myeloid Leukemia through Integrative Bioinformatics Analysis and Clinical Validation

**DOI:** 10.1155/2021/5531736

**Published:** 2021-03-19

**Authors:** Jie Wang, Yun Wu, Md. Nazim Uddin, Jian-ping Hao, Rong Chen, Dai-qin Xiong, Nan Ding, Jian-hua Yang, Jian-hua Wang, Xuan-sheng Ding

**Affiliations:** ^1^School of Basic Medicine and Clinical Pharmacy, China Pharmaceutical University, Nanjing 211198, China; ^2^Department of Pharmacy, First Affiliated Hospital of Xinjiang Medical University, Urumqi 830011, China; ^3^Department of General Medicine, First Affiliated Hospital of Xinjiang Medical University, Urumqi 830011, China; ^4^Department of Hematology, First Affiliated Hospital of Xinjiang Medical University, Urumqi 830011, China

## Abstract

Acute myeloid leukemia (AML) is a type of hematological malignancy with diverse genetic pathogenesis. Identification of the *miR-93-5p* targeted pathogenic markers could be useful for AML diagnosis and potential therapy. We collected 751 *miR-93-5p* targeted and AML-related genes by integrating the results of multiple databases and then used the expression profile of TCGA-LAML to construct a coexpression function network of AML WGCNA. Based on the clinical phenotype and module trait relationship, we identified two modules (brown and yellow) as interesting dysfunction modules, which have a significant association with cytogenetics risk and FAB classification systems. GO enrichment and KEGG analysis showed that these modules are mainly involved with cancer-associated pathways, including MAPK signal pathway, p53 signal pathway, JAK-STAT signal pathway, TGF-beta signaling pathway, mTOR signaling pathway, VEGF signaling pathway, both associated with the occurrence of AML. Besides, using the STRING database, we discovered the top 10 hub genes in each module, including *MAPK1, ACTB, RAC1, GRB2, MDM2, ACTR2, IGF1R, CDKN1A, YWHAZ,* and *YWHAB* in the brown module and *VEGFA, FGF2, CCND1, FOXO3, IGFBP3, GSF1, IGF2, SLC2A4, PDGFBM,* and *PIK3R2* in the yellow module. The prognosis analysis result showed that six key pathogens have significantly affected the overall survival and prognosis in AML. Interestingly, *VEGF* with the most significant regulatory relationship in the yellow modules significantly positively correlated with the clinical phenotype of AML. We used qPCR and ELISA to verify *miR-93-5p* and *VEGF* expression in our clinical samples. The results exhibited that *miR-93-5p* and *VEGF* were both highly expressed in AML.

## 1. Introduction

Acute myeloid leukemia (AML) is one of the most common hematological malignancies in adults and is characterized by clonal expansion of abnormally differentiated blasts of a myeloid lineage [1]. Usually, patients who are initially diagnosed with AML are often accompanied by severe clinical symptoms [2]. So, the early untreated disease progresses of AML is very rapid, and the mortality rate is also very high [3]. In recent years, with the rapid development of molecular biology and other technologies, the pathogenic genes of AML have been continuously discovered, and the development and clinical exploration of new drugs targeting AML have also been accelerated [4–6]. However, it is established that the treatment outcome of patients with AML is still not optimistic currently, and the 5-year survival rate of patients is only maintained at approximately 30% [7]. At present, AML has the characteristics of low cure rate, poor prognosis, and high rate of relapse, so it is imperative to find new biomarkers and targeted therapeutic molecules.

For nearly two decades, studies have found that the noncoding RNAs are closely related to tumor occurrence and development [8]. It plays an essential role as “oncogenes” or “tumor suppressor genes,” but the specific regulatory mechanism is still unclear entirely [9]. MicroRNAs are a noncoding single-stranded RNA class with a length of approximately 19–22 nucleotides encoded by endogenous genes [10]. It was stated that miRNA is involved with various types of critical biological processes in the human body through posttranscriptional gene expression regulation [11]. Studies have increasingly reported that miRNAs' abnormal expression is related to various tumors in the body [12]. There is also a close association between AML and several microRNAs [13, 14]. So, in-depth research on the miRNAs' function will have significant scientific significance and clinical value in AML.

*MiR-93* belongs to the *miR-106b-25* cluster, which can play an essential role in aging, hyperglycemia, and osteoblast calcification [15]. Recently, studies have demonstrated that *miR-93-5p* could participate in the occurrence, development, and drug resistance of numerous types of tumors [16–18]. Meanwhile, *miR-93-5p* can act as an oncogene by promoting angiogenesis. It can suppress integrin-*β*8 expression, which is ultimately associated with the tumor, and overexpression of *miR-93* is associated with cell spreading, growth, migration, and tube formation of cancer cells [19]. The highly expressed *miR-93-5p* has been found in other types of tumors such as neuroblastoma, non-small-cell lung cancer, and lacrimal adenoid cystic carcinoma [20–22]. Furthermore, increased expression of *miR-93-5p* is associated with the poor prognosis of gastric cancer and squamous cell carcinoma of the head and neck [23, 24]. Still, researchers have found a close association between AML and abnormal expression of *miR-93-5p*. It can promote tumor growth, blood vessel formation, and cell proliferation in AML. Also, the expression levels of *miR-93* are closely associated with *VEGF* formation in AML. These studies proved that *miR-93* is associated with several cancers, including AML [25]. However, the specific molecular mechanism that *miR-93-5p* participates in AML in pos-transcriptional regulation still needs further study. Therefore, the current study aimed to determine the expression of *miR-93-5p* and its targeted genes in AML based on integrative bioinformatics analysis and clinical validation.

## 2. Materials and Methods

### 2.1. Data Resources

Firstly, to collect the target genes of *miR-93-5p*, we used the miRNet [26] (https://www.mirnet.ca/miRNet/home.xhtml) to obtain *miR-93-5p* target genes from miRTarBase v8.0 [27], TarBase v8.0 [28], and miRecords [29] database. Thus, we got 2624 genes that were the targets of *miR-93-5p*. Moreover, we obtained a gene set that contains 985 genes that closely interact with the pathogenesis of AML from the National Center for Biotechnology Information (NCBI-Gene) database [30] and the Human Online Mendelian Genetic (OMIM) database [31]. Then, we searched in the protein interaction (STRING) database [32]. We further expanded to 3900 genes that are defined as AML-related genes in this study. Through a comprehensive comparative analysis in the expression profiling of AML from The Cancer Genome Atlas (TCGA) database (*n* = 151) [33], we have initially constructed an expression matrix including miR-93-5p targeted and AML-related potential pathogenic genes.

**Figure 1:**
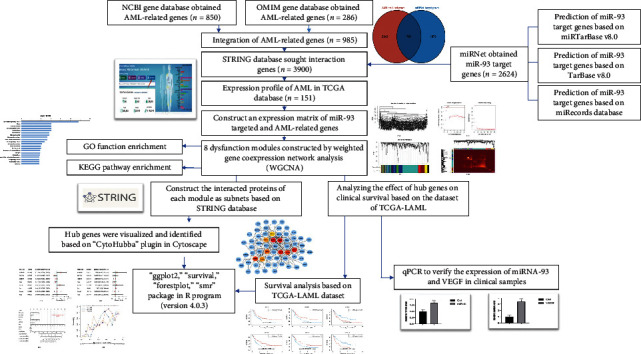
The overall analysis process of the present study.

Furthermore, we constructed a coexpression gene network in AML-related genes profile based on weighted gene coexpression network analysis (WGCNA) [34]. We analyzed the hub genes as key pathogenic markers in dysfunctional modules. Besides, the clinical data of TCGA-LAML was downloaded from TCGA to investigate the survival prognosis of key pathogenic markers. The flowchart of the analysis procedure in the previous study is shown in Figure 1.

### 2.2. Coexpression Recognition Module Based on Relevant Network Analysis

WGCNA is a newly developed method that aims to identify gene modules cooperatively expressed by characteristic genes, explore the association between gene networks and phenotypes of interest, and identify critical genes in a specific phenotype [35]. This new mathematical method is different from previous biological experimental processes. It is an effective way to identify new essential genes involved in AML and discover potential predictive and diagnostic indicators for AML patients. In this study, we used the WGCNA package for constructing the coexpression network of potential pathogenic genes in AML. In the construction process, we chose the unsigned coexpression network, selecting the power of *β* = 5 as the soft threshold to ensure the connections between genes in the network and obey the scale-free network distribution. The minimal number of genes in an individual module is set as 20.

Finally, the hierarchical clustering tree was constructed through the correlation coefficients between genes, and the similarity coexpression module was shown as the topological overlap matrix (TOM). Besides, to determine the correlation between clinical phenotype and modules, we used the person method to calculate the relationship between module eigengenes and the phenotypes of AML.

### 2.3. GO Enrichment and KEGG Analyses of the Dysfunctional Modules

To explore the genes' biological functions and signal pathways in dysfunctional modules, we used the GSEA [36] to perform the gene ontology (GO) and the Kyoto Encyclopedia of Genes and Genomes (KEGG) analysis for interested functional modules. The GO terms and KEGG pathways were confirmed as an enrichment when FDR <0.05.

### 2.4. Identification of Module Hub Genes in Dysfunctional Modules

The biomarker is helpful for the diagnosis, staging, or classification of diseases. Based on WGCNA, we have established a coexpression network of AML-related genes, and we use the Cytoscape (version 3.8.0) for screening the module hub genes [32]. Then, the “CytoHubba” [37] plugins of Cytoscape (version 3.8.0) were used to detect and visualize the hub genes of the interested functional modules. The hub genes were screened with the top 10 degrees in dysfunctional modules.

### 2.5. Analyzing the Effect of Hub Genes on Clinical Survival Based on the Dataset of TCGA-LAML

To analyze the prognosis value of screened hub genes in AML patients, the clinical information was obtained from the profile of TCGA-LAML (https://portal.gdc.cancer.gov/) in January 2020. The difference between the two groups was compared using KM survival analysis and log-rank.

For the Kaplan–Meier curve analysis, the hazard ratio (HR) with 95% confidence interval (CI) and the *P* value was derived from log-rank tests and univariate Cox proportional regression [38]. The analysis methods and packages were executed using the R program version 4.0.3 (The R Foundation for Statistical Computing, 2020). *P* < 0.05 was considered statistically significant.

The univariate and multivariate Cox regression analysis was used to identify the significant variables to establish the nomogram [39]. The “forestplot” package in the R program was used for displaying the forest plot of the *P* value, HR, and 95% CI between each variable. The multivariate Cox proportional hazard analysis results were used to develop a nomogram for predicting the 3-year overall recurrence. The nomogram provided a graphical contribution of the factors, which can be used to estimate the risk of recurrence for an individual patient by the points associated with each risk factor through the “rms” R package.

### 2.6. Verifying the Expression of *miRNA-93* and *VEGF* in Clinical Samples

To verify the expression of two molecules from the most significant modules that are still not clearly defined, we have validated the expression of *miR-93-5p* and *VEGF*. Bone marrow samples from 18 patients who were newly diagnosed with AML were collected at our institution between January 2019 and September 2019. Diagnose AML according to the classification criteria of the World Health Organization MICM (morphology, immunology, cytogenetics, and molecular biology) [40]. The inclusion criteria were as follows: the bone marrow blasts count of ≥20%. The exclusion criteria were as follows: (1) age at diagnosis <18 years and (2) follow-up information not complete. There were six male patients and 12 female patients, ranging from 20 to 79 years old. Histologically, two patients had M1, nine patients had M2, one patient had M3, two patients had M4, and four patients had M5. The bone marrow samples were collected in anticoagulated tubes before treatment. The leukemic cells were isolated by using 1.077 g/mL Ficoll-Isopaque (Pharmacia). The proportion of leukemic cells was estimated using May–Grünwald–Giemsa-stained cytocentrifuge preparations and light microscopy. The cell samples selected for analysis contained at least 90% blasts after separation. Each sample containing 2 to 10 million cells was stored in TRIzol (Invitrogen, Carlsbad, CA, USA) and frozen at −80°C as soon as possible. The clinical information of the patient included in this study was collected, with the last follow-up on September 30, 2019. The mononuclear cells were isolated from the peripheral blood of two anonymized healthy volunteers as control samples.

cDNA was generated using the Reverse Transcription Kit (Foregene, Chengdu, China). The expression levels of *miRNA-93* were quantified using SYBR Green Master Mix (SYBR GREEN, Beijing, China), and the *U6* gene was used as an internal control. The following primers were used: *miR-93-5p*, 5′-CAAAGTGCTGTT CGTGCAGGTAG -3′ and U6 5′-GGATGACACGCAAATTCGTGAAGC-3′. qRT-PCR was performed on ViiATM 7 System software (Thermo Fisher Scientific, ABI7500, USA). The results were normalized to the expression of the *U6* gene and are presented as the fold change (2^−ΔΔ*CT*^). *VEGF* protein expression by bone marrow mononuclear cells was quantified for media samples collected from different experiments using *VEGF* ELISA kit per manufacturer protocol (human vascular endothelial growth factor ELISA Kit, Wuhan, China).

## 3. Results

### 3.1. Data Processing

**Figure 2:**
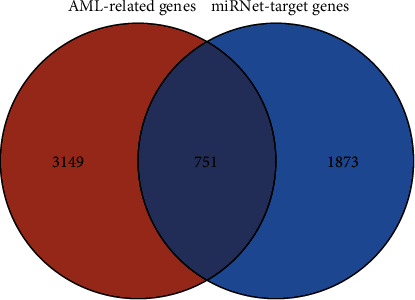
Venn diagram of *miR-93-5p* target genes and AML-related genes.

To systematically study the critical role of *miR-93-5p* regulatory target genes in the pathogenesis of AML, we collected a large number of results from multiple online databases. First, we obtained 2624 target genes of *miR-93-5p* from the miRNet, and these genes were predicted by three commonly used databases (miRTarBase v8.0, TarBase v8.0, and miRecords database). Second, to further screen these target genes related to AML, we sought to obtain 3900 AML-related genes from NCBI-Gene and OMIM databases. Then, we screened 751 interact genes between *miR-93-5p* target genes and AML-related genes to determine the essential regulatory functions of *miR-93-5p* and its target genes in AML. The Venn diagram is shown in Figure 2.

### 3.2. The Coexpression Network of *miR-93-5p* Target Genes in Acute Myeloid Leukemia Was Constructed Based on the Comprehensive Analysis

**Figure 3:**
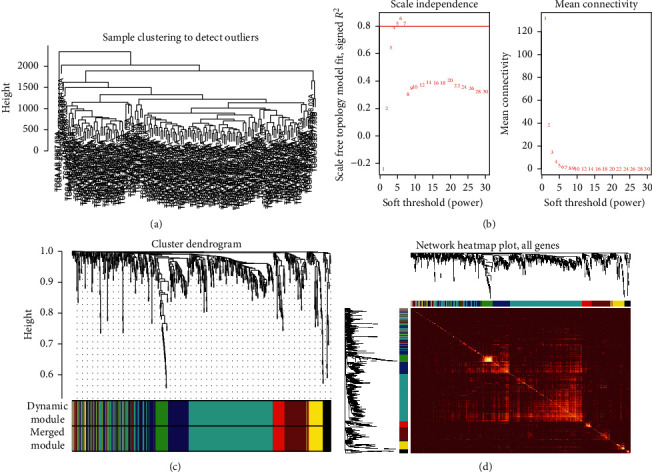
Coexpression relationship of related genes in acute myeloid leukemia clustered into modules. (a) Sample clustering to detect outliers in AML samples. (b) Cluster analysis of the scale-free topology model fit index for soft threshold powers. (c) Heat map of the expression of modular genes in AML samples. It was shown that the expression behavior of 8 modules in 151 AML samples was significantly consistent. (d) Clustering into eight modules based on differential gene coexpression relationship. One-color represents a module. AML: acute myeloid leukemia.

**Figure 4:**
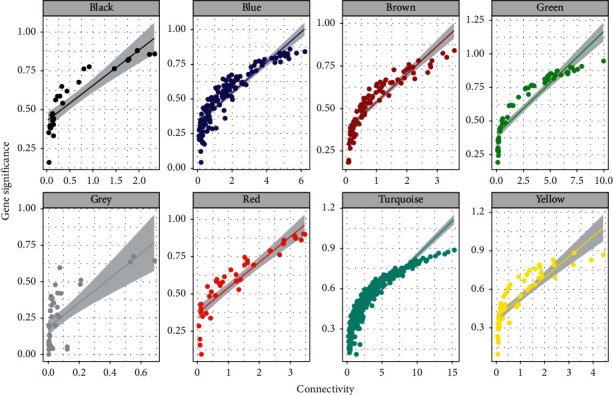
The relationship between the connectivities of the modules in each module.

**Figure 5:**
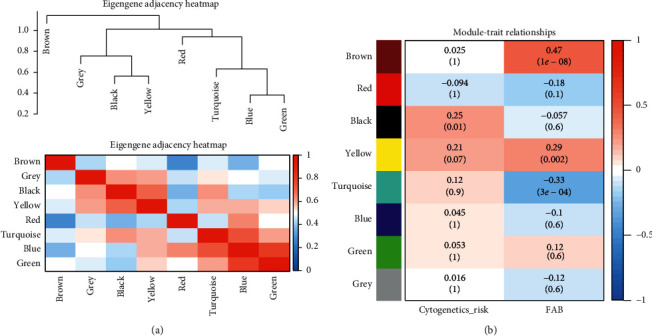
Heatmap of the clustering relationship of eigengenes adjacencies and module trait. (a) The clustering relationship between different modules. The upper part is the clustering tree between modules, and the lower part is the clustering heatmap between the modules. Red represents the closer the similarity, and blue represents the farther the similarity. (b) The clustering relationship between disease characteristics and modules. Red represents an opposite relationship, and blue represents a negative relationship.

In the current research, to match and construct an expression matrix of *miR-93-5p* targeting AML-related genes, we downloaded the RNA-seq profile of 151 AML samples from the TCGA database. The clinical information is represented in Table 1. In the process of network construction, we adopt a soft threshold (*β* = 5) to ensure the stability of the scale-free network, and we established eight coexpressed gene modules. The clustering of module gene expression behaviors in AML is shown in Figure 3. The relationship between the modules' connectivity and the gene's significance is as shown in Figure 4. The eigengene adjacency heatmap and module trait relationships are shown in Figure 5.

### 3.3. Correlation between Clinical Phenotype and Modules

**Figure 6:**
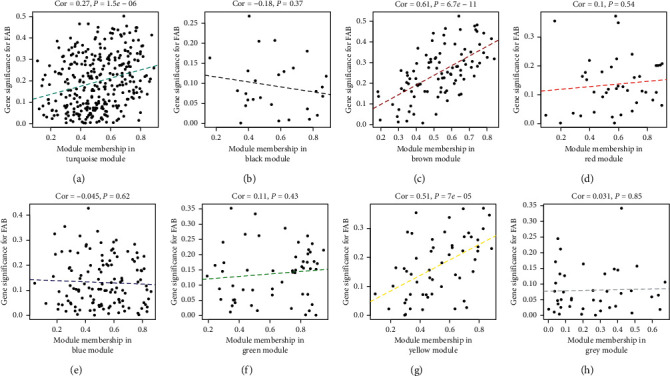
The correlation of FAB classified subtype of AML and dysfunction modules. The brown and yellow modules are significantly associated with FAB classified subtype (*P* < 0.05).

**Figure 7:**
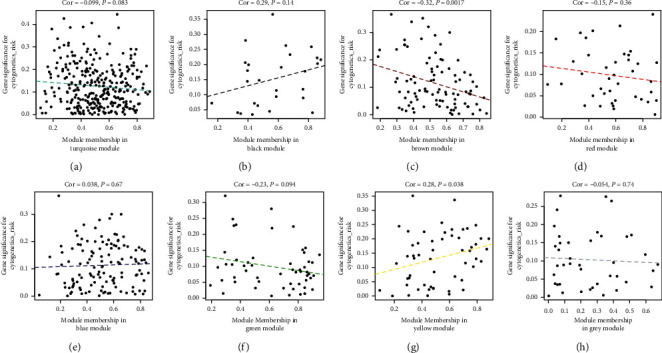
The correlation of cytogenetics risk of AML and dysfunction modules. The brown module is negatively associated with cytogenetics risk (*R*^2^ = −0.32, *P* < 0.05), and the yellow module is oppositely associated with cytogenetics risk (*R*^2^ = 0.28, *P* < 0.05).

We found that two modules are significantly correlated with the FAB classified subtypes and cytogenetic risks [41]. According to our finding, both the brown and yellow modules are positively associated with FAB classified subtype of AML (*R*^2^ = 0.61 and *R*^2^ = 0.51) (Figure 6). Furthermore, we also observed a positive correlation between the yellow module and cytogenetic risks (*R*^2^ = 0.28), and brown modules were negatively correlated with cytogenetic risks (*R*^2^ = -0.32) (Figure 7). What is noteworthy is that FAB morphological classification and cytogenetic risk are closely associated with the progress of AML, so it is necessary to identify further the biological functions and critical genes of these two modules.

### 3.4. Identification of Functional Modules and Pathways That Are Involved in the Pathogenesis of AML

**Figure 8:**
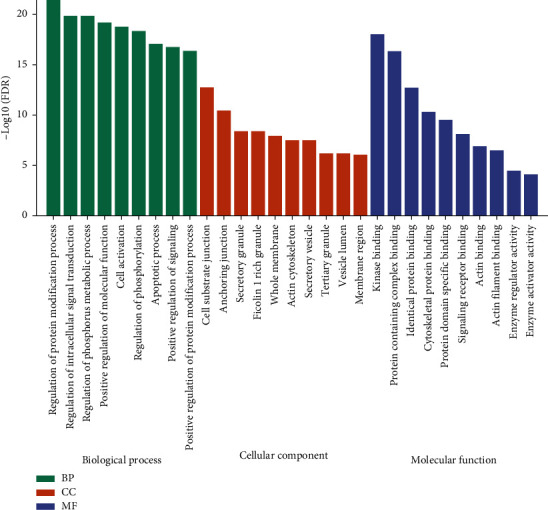
The significant GO enrichment results of brown module genes.

**Figure 9:**
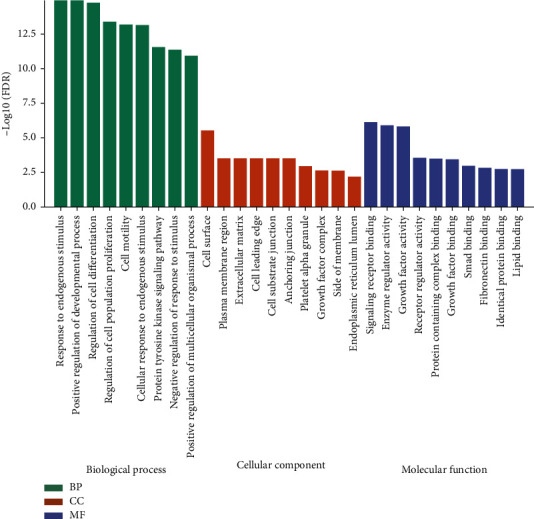
The significant GO enrichment results of yellow module genes.

**Figure 10:**
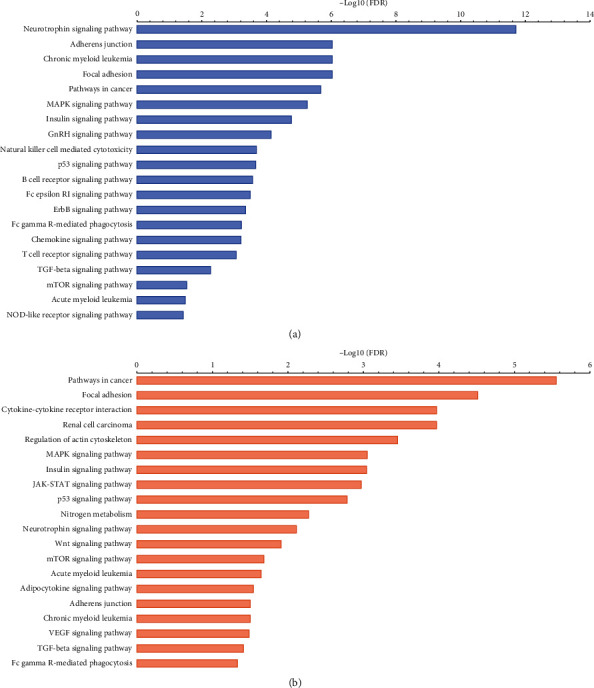
KEGG pathway analysis of two modules. (a) The results of KEGG pathways analysis of the brown module. (b) The results of KEGG pathways analysis of the yellow module.

As the two modules (yellow and brown) have been observed with the highest correlated modules with AML subtype and cytogenetic risk, we performed the GO enrichment on characteristic genes in these two modules, including biological process (BP), cellular component (CC), and molecular function (MF). The mainly enriched terms in the brown module were regulation of protein metabolic and modification process, regulation of intracellular signal transduction, regulation of phosphorus metabolic process, and positive regulation of molecular function. The significant GO terms of the brown module are visualized in Figure 8. Besides, the genes in the yellow module were mainly enriched in response to endogenous stimulus, positive regulation of the developmental process, and cell differentiation and proliferation regulation. The result of GO enrichment of the yellow module is visualized in Figure 9. The KEGG pathway analysis shows that brown and yellow modules are mainly involved pathways in cancer, neurotrophin signaling pathway, focal adhesion, adherens junction, MAPK signaling pathway, insulin signaling pathway, p53 signaling pathway, TGF-beta signaling pathway, mTOR signaling pathway, VEGF signaling pathway, and these signaling pathways involved in the occurrence, invasion, and metastasis of AML (Figure 10).

### 3.5. Identification of Hub Genes in Coexpression Modules

**Figure 11:**
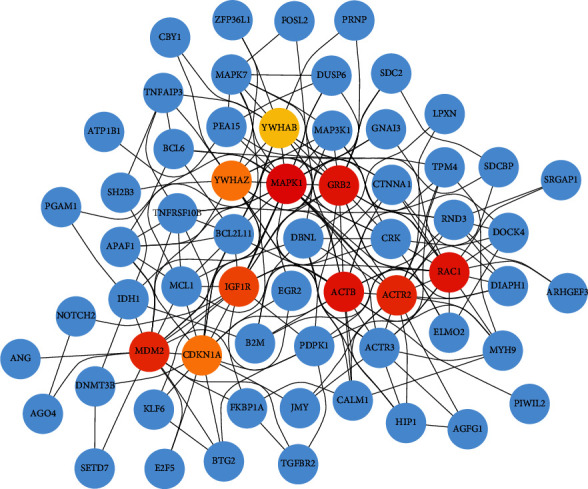
The top 10 hub genes in the brown module. The interaction of top ten hub genes with other genes in the brown module. Dark red to yellowish nodes indicate the hub genes. The hub nodes with dark red indicating the greater degree of interaction and Yellowish color hub nodes indicate the comparatively lower number of interactions. Blue nodes are other genes that are interacting with hub genes.

**Figure 12:**
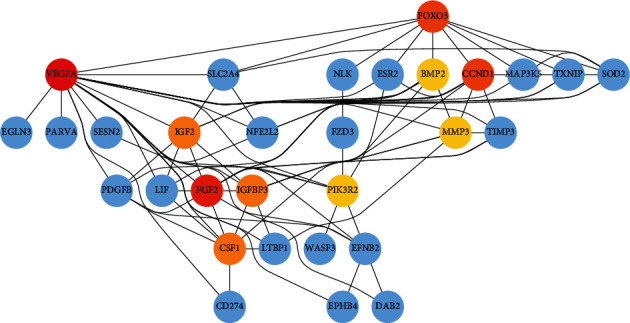
The top 10 hub genes in the yellow module. The interaction of top ten hub genes with other genes in the brown module. Dark red to yellowish nodes indicate the hub genes. The hub nodes with dark red indicating the greater degree of interaction and Yellowish color hub nodes indicate the comparatively lower number of interactions. Blue nodes are other genes that are interacting with hub genes.

Our research found that the brown and yellow functional modules are composed of many potential genes, and these genes have significant interaction regulation relationships among different available modules. Therefore, we constructed the protein-protein interaction (PPI) network in brown (83 nodes and 243 edges) and yellow modules (41 nodes and 103 edges) based on the STRING database. Then, we screened the top 10 hub genes in these two modules separately, including *MAPK1, ACTB, RAC1, GRB2, MDM2, ACTR2, IGF1R, CDKN1A, YWHAZ,* and *YWHAB* in the brown module (Figure 11), and *VEGFA, FGF2, CCND1, FOXO3, IGFBP3, GSF1, IGF2, SLC2A4, PDGFBM,* and *PIK3R2* in the yellow module (Figure 12). These genes play essential regulatory roles in the modules and have been identified as critical roles in the pathogenesis of AML.

### 3.6. The Affection of Hub Genes on Survival in AML Patients

**Figure 13:**
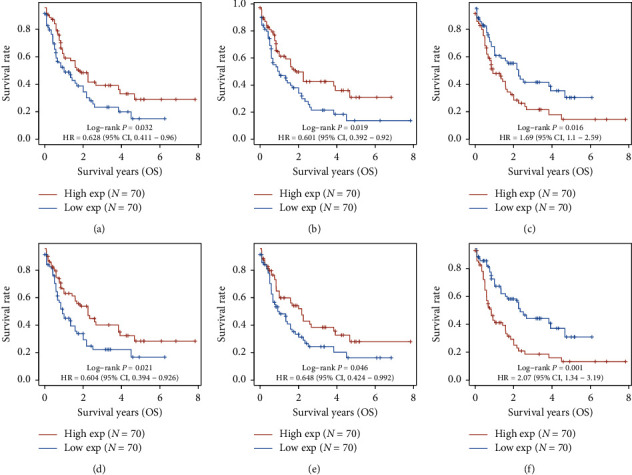
The Kaplan–Meier survival analysis of the gene signature. (a) IGF2, (b) IGFBP3, (c) ACTR2, (d) SLC2A4, (e) IGF1R, and (f) YWHAB.

**Figure 14:**
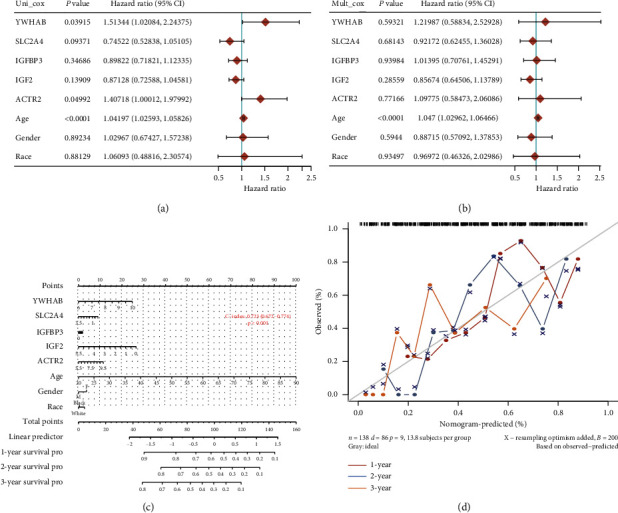
The results of Cox regression and nomogram analysis in hub genes. (a) Hazard ratio and *P* value involved in univariate Cox regression and the parameters of the hub genes. (b) Hazard ratio and *P* value involved in multivariate Cox regression and some parameters of the hub genes. (c) Nomogram to predict the 1-year, 2-year, and 3-year overall survival based on AML patients' characteristics and critical gene expression. (d) Calibration curve for the overall survival nomogram model in the discovery group. A dashed diagonal line shows the ideal nomogram, and the blue line, red line, and orange line represent the 1-year, 2-year, and 3-year observed nomograms.

To verify the screened key pathogens' clinical prognosis value, we demonstrated the effects of hub genes on the AML prognosis from the TCGA database. The result shows that six hub genes significantly impact the overall survival by using a *log-rank* test (*P* < 0.05). Highly expressed *ACTR2* and *YWHAB* are associated with poor prognosis. Low expression of *IGF2, IGFBP3, SLC2A4,* and *IGF1R* is related to poor prognosis contrarily (Figure 13). Besides, the result of nomogram analysis shows that these hub genes both affect AML's overall survival, and we could predict the 1-year, 2-year, and 3-year survival based on patient characteristics and the hub genes expression level (Figure 14).

### 3.7. Validation of the Expression of Key Genes in Clinical Samples

**Figure 15:**
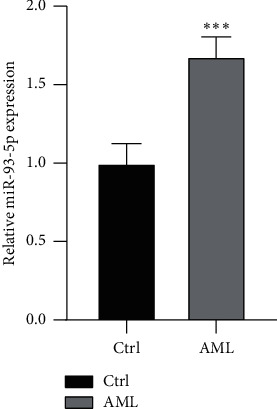
The different expression of *miR-93-5p* between AML and healthy group. Ctrl represents the healthy group.

**Figure 16:**
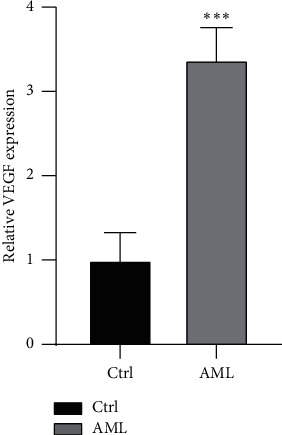
The different expression of *VEGF* between AML and healthy group. Ctrl represents the healthy group.

In this study, the *miR-93* targeted genes with significant correlation with AML were identified. We noticed that the *miR-93-5p* targeted genes had not been extensively studied in AML, and the considerable impact of *miR-93-5p* target gene networks on pathogenesis in AML is still unclear. Thus, to further verify the specific characteristic genes found in this study, we selected the genes with the most significant regulatory relationship among the yellow modules that are significantly positively correlated with the clinical phenotype of AML. Thus, we verified the expression of *miR-93-5p* and *VEGF* in our clinical samples. The results indicated that *miRNA-93-5p* is highly expressed in AML, which is significantly higher than in the healthy (Figure 15). Besides, we also found that *VEGF* is highly expressed in AML (Figure 16). These results might provide us with novel insights for further molecular phenotype and function mechanism research.

## 4. Discussion

Acute myeloid leukemia (AML) is a highly aggressive malignant tumor caused by abnormal cloning of hematopoietic stem cells [42]. Cytogenetic abnormalities have been identified as diagnostic and prognostic markers [43]. In AML with different genetic characteristics, miRNAs have differential expression profiles, and this differential expression profile can be used as a basis for clinical diagnosis and prognosis of AML [44]. The carcinogenic mechanism of some miRNAs that are abnormally expressed in leukemia has been elucidated [45]. It is expected that drugs targeting these miRNAs will be developed in the future to provide new targets for the treatment of leukemia.

MicroRNA-93 is a member of the *miR-106b-25* cluster, located in intron 13 of the *MCM7* gene [46]. It is a new type of miRNA that is highly expressed in a variety of human malignancies. Increasing evidence indicates that abnormal expression of *miR-93-5p* involves many kinds of human tumors [47, 48]. The dysregulation of *miR-93-5p* is associated with the development of multidrug resistance in various types of cancer [20, 49]. Recently, research has reported *miR-93-5p* was abnormally expressed in AML, and the abnormal expression of *miR-93-5p* may affect the prognosis of AML [50]. However, the exact mechanism of *miR-93-5p* in AML is still unclear. Therefore, to fully explore the specific mechanism of *miR-93-5p* regulating target genes to mediate in the pathogenesis of AML, we adopted comprehensive bioinformatics research. In this study, we obtained 2,624 *miR-93-5p* target genes through the prediction of target genes from multiple online databases. At the same time, to further determine these *miR-93-5p* target genes are closely associated with the pathogenesis of AML, we collected a gene list related to AML through NCBI-GENE and OMIM. Then, we obtained 751 interacted genes between *miR-93-5p* target genes and AML associated genes. Besides, based on the RNA-seq profile of TCGA-LAML, we constructed an expression matrix for WGCNA analysis. Our new ideas about collecting disease-related genes and gene cluster modularization may provide a feasible research method for other diseases like AML that lack strict controls. As the result of WGCNA, we conducted eight dysfunction modules to characterize the pathogenesis of AML. Interestingly, we found that two modules were significantly correlated with the phenotype of AML. Furthermore, GO analysis shows that these two modules are involved in protein metabolism and modification, intracellular signal transduction, and cell differentiation and proliferation regulation. KEGG pathway analysis also indicates that these two modules were mainly involved in the cancer pathways, such as MAPK signaling pathway, insulin signaling pathway, p53 signaling pathway, TGF-beta signaling pathway, mTOR signaling pathway, VEGF signaling pathway, and other different pathways that are closely connected with the pathogenesis of AML [51, 52].

As we have found in our research, the hub genes with core regulatory roles in brown and yellow modules were further identified. Using connectivity to calculate the relationship between module genes, we screened ten hub genes in each module. *VEGF* has been a core regulatory position in the yellow module, and *MAPK1* also acts as a core regulatory role in the brown module. Vascular endothelial growth factor (*VEGF)* is an essential factor in promoting angiogenesis in vivo, promoting the proliferation, differentiation, and infiltration of tumor cells [53]. It has been proved that *VEGF* could lead to an increase of leukemic cells, and patients with higher expression levels of *VEGF* are more likely to have poor prognoses [54]. Studies have found that AML patients with higher expression of *VEGF* are more likely to relapse [54, 55]. As far as we have known, angiogenesis plays an essential role in the bone marrow microenvironment, which leads to the pathogenesis, metastasis, and infiltration of AML. The *VEGF/VEGFR* complex's critical regulatory part in angiogenesis may be the key to studying the pathogenesis of AML [56]. Furthermore, the current studies have found that *VEGF* may affect the pathogenesis and treatment of AML in three forms: higher levels of *VEGF* can be detected in the BMSCs of leukemia patients, which resists the apoptosis of leukemia cells by chemotherapeutics through autocrine pathways and promotes leukemia Cell resistance; *VEGF* can stimulate endothelial cells to secrete growth factors and reversely bind to receptors on leukemia cell membranes, thereby promoting vascular endothelial proliferation and secreting cytokines that encourage cell leukemia proliferation and indirectly promote the pathogenesis of leukemia through the paracrine pathway; *VEGF* can also promote the expansion of leukemia stem/progenitor cells [57]. Therefore, research on the *VEGF* signaling pathway's downstream regulatory mechanism will guide the development of new treatments for AML.

In addition, we verified the expression of *VEGF* and *miR-93-5p* in our clinical samples. The result shows that these two regulators are both highly expressed in AML compared to those in the healthy group. *miRNA-93* is a member of the *miR-106b-25* cluster, which plays an essential regulatory function in promoting oncogenes' expression and inhibiting the expression of apoptotic proteins [58]. Studies have shown that the overexpression of *miRNA-93* can promote osteosarcoma proliferation and angiogenesis [59]. Besides, experiments on leukemia cells in vitro found that the high expression of *miRNA-93* is closely related to the increase of *VEGF*. The proliferation of *VEGF* expression level is also clinically associated with AML patients' prognosis [50]. These related results further reflect the potential biological functions of the two biomarkers. Their regulatory relationship could be an essential molecular mechanism that induced tumor cell proliferation and microangiogenesis and even mediates the pathogenesis of AML [60]. Unfortunately, we have not yet found research reports on the exact mechanism of the specific regulatory relationship between *miR-93-5p* and *VEGF*. However, after predicting target genes through the TargetScan Human database (http://www.targetscan.org), we discovered that *miRNA-93* might be targeting hypoxia-inducible factor 1*α* (*HIF-1α*) affecting the expression of *VEGF* [61]. At the same time, studies have found that the *miRNA-93* consensus sequence also exists in the 3′UTR region of *VEGF* mRNA, which inspires our next research: *miRNA-93* may mediate the occurrence development of AML by regulating the expression of *HIF-1α/VEGF* [22]. However, the cell phenotype produced by *miR-93* regulating the expression of *HIF-1α/VEGF* in AML and whether its expression characteristics are related to the treatment and prognosis of AML are scientific issues worthy of further study.

*MiR-93-5p* may be associated with the translational signaling of AML and a broader spectrum of hematological malignancies. The mechanistic study revealed that *miR-93* was found to inhibit the phosphorylation of AKT (pAKT) [62]. In contrast, *miR-93* promoted the proliferation, invasion, progression, and metastasis of cancer cells through activation of PI3K/AKT signaling [16, 63, 64]. Also, overexpression of *miR-93* has associated with drug resistance in cancer cells through the miR-93/PTEN/AKT signaling pathway [65]. Besides, the PI3K-Akt-mTOR signaling pathway is upregulated in AML cells, ultimately contributing to metabolic reprogramming of AML [66]. Similarly, dysregulated mammalian target of rapamycin (mTOR) promotes AML. mTOR plays a central role in AML and a broader spectrum of hematological cancers. It was found that the rapamycin derivatives inhibit AKT signaling in primary AML cells both in vitro and in vivo, supporting the therapeutic potential of mTOR inhibition strategies in leukemias and multiple myeloma dissemination and angiogenesis [67, 68]. Interestingly, we also got the mTOR pathway is enriched in the dysfunctional modules (Figure 10) of AML. It was stated that mTOR is implicated in leukemic cell growth, tumor-associated angiogenesis, and VEGF expression in AML [69, 70]. The persistently secreted *VEGF* from myeloid cells and the elevated levels were detected in the AML microenvironment [56], which is one of the important pathophysiological processes. Since inhibition of mTOR complex 2 restrains tumor angiogenesis, mTORC2 is an advanced stage of a translational application being a theragnostic target [68] and also promotes cancer via the formation of new vessels that are essential for the growth and energy production of cancer cells. Altogether, *miR-93* may be associated with regulating the miR-93/AKT/mTOR/VEGF pathway in AML, and clinical investigation will be warranted. However, our findings still need more clinical samples and broader prospective studies to validate further.

## 5. Conclusions

In summary, our work provides comprehensive and novel insights into the pathogenesis of AML. By constructing a coexpression module of *miR-93-5p* targeted and AML-related genes and verifying the expression of clinical samples, we initially explored the molecular mechanism of *miR-93-5p* targeting and regulating *VEGF*-mediated pathogenesis of AML. Further, we laid a theoretical foundation for the research on targeting *miR-93-5p* to treat AML.

## Figures and Tables

**Table 1:** Clinical information of the patient with AML from TCGA.

Patient characteristic	Total
Age	54.17 ± 17.07
Gender	
Male	83
Female	68
Leukocyte	35.02 ± 41.34
Monocyte	11.72 ± 15.13
Hemoglobin	9.47 ± 1.62
Platelet	64.38 ± 53.13
FAB classification systems	

Mo	15
M1	35
M2	38
M3	15
M4	29
M5	15
M6	2
M7	1
Not classified	1
Cytogenetics risk	

Unclassified	2
Favorable	31
Intermediate/normal	82
Poor	36

Note: FAB classification systems, French-American-British classification systems.

## Data Availability

The data used in this study can be available as follows: TCGA-AML cohort is downloaded from the The Cancer Genome Atlas (TCGA) database (https://portal.gdc.cancer.gov/). The National Biotechnology Information Center Gene (NCBI-Gene) database (https://www.ncbi.nlm.nih.gov/gene) and the Human Online Mendelian Genetic (OMIM) database (https://omim.org/) were utilized for collecting the set of genes.

## Supplementary Materials

Supplementary Materials The graphical abstract of the manuscript is included in the supplementary file.
