# Supplementary figures and images for: Identification of MiR-93-5p Targeted Pathogenic Markers in Acute Myeloid Leukemia through Integrative Bioinformatics Analysis and Clinical Validation
Source: J Oncol. 2021 Mar 19;2021:5531736. doi: 10.1155/2021/5531736 (PMC8004384; doi:10.1155/2021/5531736)

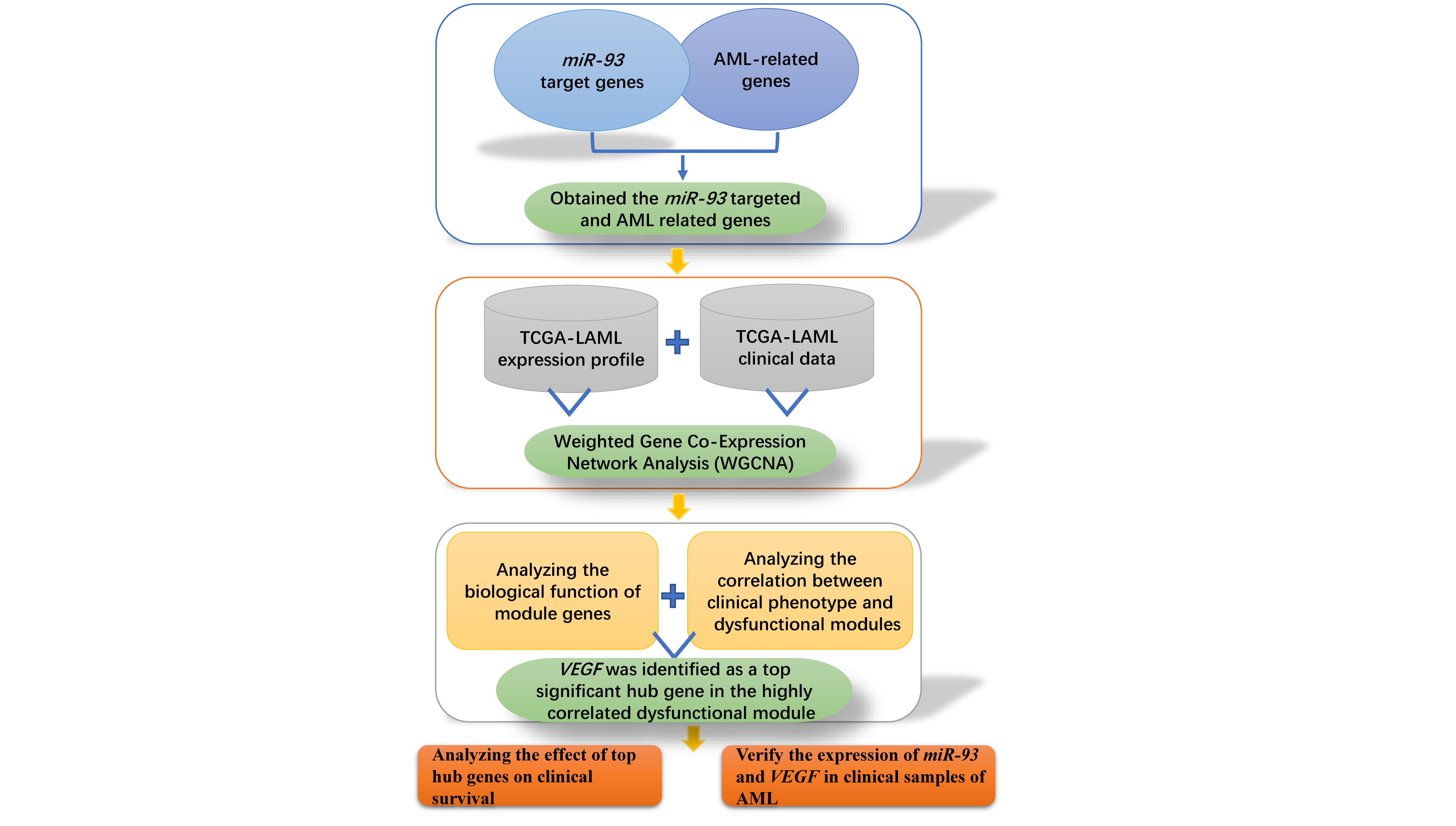

Supplement: Supplementary Materials — The graphical abstract of the manuscript is included in the supplementary file. [file 5531736.f1.png]
